# Spontaneous Resistance of *Erwinia amylovora* Against Bacteriophage Y2 Affects Infectivity of Multiple Phages

**DOI:** 10.3389/fmicb.2022.908346

**Published:** 2022-08-01

**Authors:** Leandra E. Knecht, Yannick Born, Cosima Pelludat, Joël F. Pothier, Theo H. M. Smits, Martin J. Loessner, Lars Fieseler

**Affiliations:** ^1^Food Microbiology Research Group, Institute of Food and Beverage Innovation, Zurich University of Applied Sciences (ZHAW), Wädenswil, Switzerland; ^2^Institute of Food, Nutrition and Health, ETH Zürich, Zurich, Switzerland; ^3^Agroscope, Plant Pathology and Zoology in Fruit and Vegetable Production, Wädenswil, Switzerland; ^4^Environmental Genomics and Systems Biology Research Group, Institute of Natural Resource Sciences, Zurich University of Applied Sciences (ZHAW), Wädenswil, Switzerland

**Keywords:** bacteriophage, spontaneous resistance, *Erwinia amylovora*, LPS (lipopolysaccharide), amylovoran

## Abstract

Broad application of antibiotics gave rise to increasing numbers of antibiotic resistant bacteria. Therefore, effective alternatives are currently investigated. Bacteriophages, natural predators of bacteria, could work as such an alternative. Although phages can be highly effective at eliminating specific bacteria, phage resistance can be observed after application. The nature of this resistance, however, can differ depending on the phage. Exposing *Erwinia amylovora* CFBP 1430, the causative agent of fire blight, to the different phages Bue1, L1, S2, S6, or M7 led to transient resistance. The bacteria reversed to a phage sensitive state after the phage was eliminated. When wild type bacteria were incubated with Y2, permanently resistant colonies (1430^*Y*2*R*^) formed spontaneously. In addition, 1430^*Y*2*R*^ revealed cross-resistance against other phages (Bue1) or lowered the efficiency of plating (L1, S2, and S6). Pull down experiments revealed that Y2 is no longer able to bind to the mutant suggesting mutation or masking of the Y2 receptor. Other phages tested were still able to bind to 1430^*Y*2*R*^. Bue1 was observed to still adsorb to the mutant, but no host lysis was found. These findings indicated that, in addition to the alterations of the Y2 receptor, the 1430^*Y*2*R*^ mutant might block phage attack at different stage of infection. Whole genome sequencing of 1430^*Y*2*R*^ revealed a deletion in the gene with the locus tag *EAMY_2231.* The gene, which encodes a putative galactosyltransferase, was truncated due to the resulting frameshift. The mutant 1430^*Y*2*R*^ was monitored for potential defects or fitness loss. Weaker growth was observed in LB medium compared to the wild type but not in minimal medium. Strain 1430^*Y*2*R*^ was still highly virulent in blossoms even though amylovoran production was observed to be reduced. Additionally, LPS structures were analyzed and were clearly shown to be altered in the mutant. Complementation of the truncated *EAMY_2231 in trans* restored the wild type phenotype. The truncation of *EAMY_2231* can therefore be associated with manifold modifications in 1430^*Y*2*R*^, which can affect different phages simultaneously.

## Introduction

Bacteriophages are viruses which exclusively infect bacteria. They pose the most abundant biological entity on Earth reaching an estimated number of 10^31^ (Casjens, 2005; Hatfull, 2008; Cobián Güemes et al., 2016) and outnumber bacteria tenfold (Brüssow and Hendrix, 2002). It has been estimated that approximatively 10^25^ phage infections occur every second (Pedulla et al., 2003). Bacteria are therefore forced to adapt to this selective pressure. The co-evolution of bacteria and phages generates a continuous arms race (Labrie et al., 2010; Samson et al., 2013).

Bacteria have several mechanisms to interfere with phage infection, which can target the process at different stages (Labrie et al., 2010; Seed, 2015). Phages recognize their host bacteria by binding to specific receptors on the host surface. These receptors can be proteins, sugar molecules or cell surface structures located either in the outer membrane (Marti et al., 2013), in capsules (Fehmel et al., 1975), or located in appendages such as pili (Guerrero-Ferreira et al., 2011) and flagella (Shin et al., 2012; Bertozzi Silva et al., 2016). By mutating or masking the targeted receptor, bacteria can protect themselves from phage infection at this early stage (Riede and Eschbach, 1986; Pedruzzi et al., 1998; Harvey et al., 2018). Another option to block phage adsorption is the production of competitive inhibitor molecules, that outcompete the phage for receptor binding (Destoumieux-Garzón et al., 2005). Bacteria can also reduce the accessibility of the receptor by secreting exopolysaccharides (EPS) covering the receptor (Forde and Fitzgerald, 2003; Scholl et al., 2005), or, if challenged with EPS-specific phages, they can reduce the amount of EPS secretion (Labrie et al., 2010). After the attachment to the host receptor, the phage injects its genome into the host cell. Phage resistance can be established by blocking DNA entry into the cell through superinfection exclusion (sie) systems (Moak and Molineux, 2000; Bebeacua et al., 2013). These systems are usually encoded by prophages to protect the lysogenized host from further phage infections (Dy et al., 2014). After successful attachment and DNA injection, the bacterial metabolism is hijacked by the phage and modified into producing and assembling new phage particles. If phage DNA reaches the cytoplasm of the host cell, a collection of intracellular defense mechanisms can act to prevent phage replication. Restriction-modification (R–M) systems recognize the incoming foreign DNA and digest it (Tock and Dryden, 2005). CRISPR/Cas systems function similarly such that incoming phage DNA is identified and cleaved. In contrast to the R–M system, they are highly specific against certain phages (Dupuis et al., 2013). While R–M and CRISPR/Cas systems can cause survival of the infected cell, the abortive infection system (Abi) is fatal. These systems are diverse in sensing and reacting to phage infection. However, once activated, the infected bacterium destroys itself thereby preventing phage proliferation (Dy et al., 2014). This sacrifice ensures survival of the surrounding bacterial population.

Phage resistance can occur spontaneously where it was observed to be either transient or permanent (Moineau, 1999; Oechslin, 2018). Permanent resistance against phages can for example involve genetic mutations. Mutations in genes that encode the receptor or are crucial for receptor biosynthesis prevent infection by phages that would have targeted the particular receptor (Nesper et al., 2000). Many studies, however, have associated phage resistance with fitness loss (Smits et al., 2011; Koskella et al., 2012). Such a fitness loss could favor the susceptible over the phage resistant state. In the case of transient resistance, the phage sensitive state will be re-established after elimination of the phage. Bacteria can for example, apply phase variation to generate transient resistance (van der Woude and Bäumler, 2004; Seed et al., 2012). Alterations of prominent structures such as capsules and flagella can be regulated by phase variation as observed in *Campylobacter jejuni* (Gencay et al., 2018) or *Salmonella enterica* (Cota et al., 2012; Kim and Ryu, 2012). Modifications in response to a particular phage can potentially entail resistance against other phages. Phages adsorbing to the same receptor will be unable to bind to a modified receptor. Consequently, phages are often applied as so called “phage cocktails”. A phage cocktail usually contains phylogenetically distinct phages targeting different receptors of the host cell surface to enhance phage treatment and to decrease the likelihood of resistance development.

*Erwinia amylovora* is the causative agent of fire blight, a plant disease affecting members of the *Rosaeceae* family (Bonn and van der Zwet, 2000; Momol and Aldwinckle, 2000; Thomson, 2000; Vanneste, 2000; Gill et al., 2003). Fire blight was previously classified as one of the ten economically most damaging plant disease (Mansfield et al., 2012). The Gram-negative bacterium has a collection of factors contributing to its pathogenicity (Piqué et al., 2015). The pathogen proliferates in the blossoms on the stigmas before entering the plant tissue. Inside the plant tissue, a biofilm is produced, which clogs the xylem vessels (Geider, 2000; Vanneste and Eden-Green, 2000; Wei et al., 2000; Koczan et al., 2011; Castiblanco and Sundin, 2016). The affected tissues start to desiccate (Vanneste and Eden-Green, 2000). At this stage, the disease spread can only be stopped by pruning of infected tissues or eliminating the entire plant. Antibiotics such as streptomycin are the most efficient treatment of fire blight during the blossoming season. The emergence of antibiotic resistant bacteria (Jones and Schnabel, 2000; Piqué et al., 2015) resulted in banning the antibiotic for agricultural application in an increasing number of countries (Russo et al., 2008). Therefore, efficient and environmentally friendly alternatives are currently under investigation. Bacteriophages were shown to have the potential to eliminate *E. amylovora* efficiently and specifically (Lehman, 2007; Svircev et al., 2010; Boulé et al., 2011; Akremi et al., 2020).

*Erwinia amylovora* specific phages Bue1 (MG973030), L1 (HQ728265), M7 (HQ728263), S2 (MG736918), S6 (HQ728266), and Y2 (HQ728264) were isolated previously from Swiss orchards (Born et al., 2011; Knecht et al., 2018). The phages are classified as members of the *Myoviridae* (M7), *Chaseviridae* (Y2), *Autographiviridae* (L1, S2), *Schitoviridae* (S6), and *Ackermannviridae* (Bue1) families (Born et al., 2011; Knecht et al., 2018). All six phages were shown to have a broad host range and are strictly virulent. The phages were tested for their phytotherapeutic effect against *E. amylovora in vitro*. Different combinations of these phages were shown to have the potential to control bacterial cell counts over a prolonged time (Born et al., 2011; Knecht et al., 2018). However, the diversity of bacteriophages infecting *E. amylovora* is much greater and novel phages have been described recently (Thompson et al., 2019; Akremi et al., 2020; Besarab et al., 2020; Kim et al., 2020).

Even though phages are an effective alternative to antibiotics, resistance development should be avoided. To prevent phage resistance development in the plant pathogens, the underlying mechanisms generating phage resistance and cross-resistance against other phages must be further investigated. This study aimed to anticipate the risk of spontaneous phage resistance by *E. amylovora* CFBP 1430 (NC_013961) against a variety of phages. In contrast to Bue1, L1, M7, S2, and S6, phage Y2 induced a permanent resistance in the bacterium. Furthermore, the permanent resistant mutant showed cross-resistance against the other phages.

## Materials and Methods

### Culture Conditions

*Erwinia amylovora* strains were cultivated in LB medium (10 g/l tryptone, 10 g/l NaCl, 5 g/l yeast extract, with or without the addition of 15 g/l agar, Laboratorios Conda S.A, Spain) at 28°C, *Escherichia coli* strains at 37°C. Ampicillin was added at a concentration of 100 μg/ml if required. In addition, strains of *E. amylovora* were also cultivated in MM2 minimal medium (1.6 g/l K_2_HPO_4_, 3.0 g/l NaCl, 10 g/l sorbitol, 0.2 mg/ml nicotic acid, 0.2 mg/ml thiamin hydrochloride, 4 mg/ml L-asparagine, and 0.205 mg/ml MgSO_4_) at 28°C under shaking for 24 h. All strains used are listed in Table 1.

**TABLE 1:** Table 1Overview of the used bacterial strains, generated mutants and applied phages.

Bacterial strains	Characterization	References
*Erwinia amylovora*		
CFBP1430	Wild type strain, isolated in France from *Crataegus* sp.; propagation strain for all phages	Zhang and Geider, 1997
CFBP1430 [pBAD18]	Empty vector control	This work
CFBP1430^*Y*2*R*^	Spontaneous permanent Y2 resistant strain	This work
CFBP1430 [pBAD18:*EAMY_2231*]	Overexpression of *EAMY_2231* from pBAD:*EAMY_2231*	This work
CFBP1430^*Y*2*R*^ [pBAD18:*EAMY_2231*]	Complementation of 1430^*Y*2*R*^ with *EAMY_2231* from pBAD:*EAMY_2231*	This work
Ea4/82	Isolated in Egypt from *Pyrus communis*; low EPS-producer	Zhang and Geider, 1997
*Escherichia coli*		
Dh5α	supE44 ΔlacU169 (φ80lacZΔM15) hsdR17 recA1 endA1 gyrA96, thi-1 relA1	Hanahan, 1983
XL1-Blue MRF’		NEB (Ipswich, United States)
**Plasmids**		
pBAD18	Complementation vector, arabinose induced pBAD promoter	Guzman et al., 1995
pBAD18:*EAMY_2231*	Complementation vector for *EAMY_2231*, arabinose induced pBAD promoter	This work
**Phages**		
Bue1	*Erwinia amylovora* specific phage (*Ackermannviridea*)	Knecht et al., 2018
L1	*Erwinia amylovora* specific phage (*Autographiviridae*)	Born et al., 2011
M7	*Erwinia amylovora* specific phage (*Myoviridae*)	Born et al., 2011
S2	*Erwinia amylovora* specific phage (*Autographiviridae*)	Knecht et al., 2018
S6	*Erwinia amylovora* specific phage (*Schitoviridae*)	Born et al., 2011
Y2	*Erwinia amylovora* specific phage (*Chaseviridae*)	Born et al., 2011

### Soft Agar Overlay and Propagation

Phages (Table 1) were propagated using the soft agar overlay method (Adams, 1959). Four milliliters molten LB + soft agar (LB medium, 4 g/l agar, 2 mM MgSO_4_, and 10 mM CaCl_2_) were supplemented with 90 μl bacterial overnight culture and 10 μl diluted phage and spread on LB agar plates to generate semi-confluent lysis of the bacteria. After overnight incubation, 5 ml SM buffer (100 mM NaCl, 8 mM MgSO_4_, 50 mM Tris-Cl, pH 7.4) were added per plate and incubated for 5 h at room temperature (RT) under shaking. The supernatant was amended with 0.5 M NaCl and incubated for 30 min at RT before centrifugation (10 min, 10,000 × g). Phages in the supernatant were then polyethylenglycol treated [10% (w/v) PEG 8000, placed in an ice bath overnight] and pelleted by centrifugation (15 min, 10,000 × g, 4°C). Then they were CsCl density gradient purified (Sambrook and Russell, 2001) and dialyzed against SM buffer for 6 h at RT (Bue1, L1, M7, S2, and S6). In the case of Y2, PEG 8000 was removed by incubating the PEG-phage mixture at RT for 1 h. The solution was then centrifuged at 5,000 × g for 10 min. Phages in the supernatant were filter sterilized (0.22 μm filter). All phages were stored at 4°C.

### Spontaneous Resistance

To identify spontaneous resistance against phages, *E. amylovora* CFBP 1430 was incubated in LB medium at 28°C under shaking over night. The next day this culture was diluted to 10^5^ CFU/ml in LB + broth (LB medium, 2 mM MgSO_4_, 10 mM CaCl_2_) and supplemented with 10^8^ PFU/ml phages. The suspension was incubated for 5 h under shaking at 28°C before a dilution series (10^–1^ to 10^–8^) was plated onto LB agar plates. After overnight incubation at 28°C, 10 colonies per phage treatment were selected and again incubated in LB medium at 28°C under shaking over night. The resulting liquid cultures were then used to determine phage resistance on LB+ soft agar overlays at 28°C. In addition, the supernatants of the overnight cultures were also tested for remaining phages. For each phage the experiment was repeated three times. The Y2 resistant mutant termed 1430^*Y*2*R*^ was further investigated.

### Phage Infectivity

To investigate the ability of different phages to infect the 1430^*Y*2*R*^ strain, 5 μl of serially diluted phages were spotted onto a LB+ soft agar lawn inoculated with 100 μl bacterial overnight culture. The infectivity of six phages (Table 1) was tested on the CFPB 1430 wild type, the empty vector control (CFPB 1430 [pBAD18]), 1430^*Y*2*R*^, 1430 [pBAD:*EAMY_2231*], and 1430^*Y*2*R*^ [pBAD:*EAMY_2231*] and their plaque forming ability was monitored. Except for the wild type, overnight cultures, soft agar and LB plates were amended with 100 μg/ml ampicillin to maintain pBAD18 plasmids and 0.2% (w/v) arabinose for pBAD18 promoter induction. The experiment was performed with three biological and three technical replicates.

### *In vitro* Infection Assay

The impact of 1430^*Y*2*R*^ on phage infectivity in liquid medium was evaluated by *in vitro* infection assays. Overnight cultures of *E. amylovora* were prepared as described above, washed twice in sterile SM buffer and OD_600 *nm*_ was adjusted to reach approximately 10^7^ CFU/ml. Subsequently, 20 μl of the washed cells were transferred to 1,960 μl LB + broth and supplemented with either 20 μl sterile SM buffer or with 20 μl phages with a concentration of 10^10^ PFU/ml. For the maintenance of the pBAD18 plasmids 100 μg/ml ampicillin was added to the cultures. For pBAD18 promoter induction 0.2% (w/v) arabinose was used. The mixtures were then added to 96 well flat bottom plates and incubated at 25°C with double orbital shaking for 24 h in a Biotek Synergy H1 Hybrid plate reader. The OD_600 *nm*_ was recorded every 30 min. The experiment was performed with three biological and three technical replicates.

### Growth Curves

Impact of the mutations on fitness of 1430^*Y*2*R*^ was investigated. Bacteria were washed twice in SM buffer and OD_600 *nm*_ was adjusted to 0.1. Cells were diluted in LB broth amended with 2 mM MgSO_4_ and 10 mM CaCl_2_ or MM2 medium (Bellemann et al., 1994) to a concentration of 10^5^ CFU/ml. Cultures were incubated at 28°C for 24 h with double orbital shaking (150 rpm) in a Biotek Synergy H1 Hybrid plate reader. The OD_600 *nm*_ was measured every 30 min. The experiment was performed with three biological and three technical replicates.

### Detached Flower Assay

To monitor virulence of strain 1430^*Y*2*R*^, a detached flower assay using fresh blossoms from 2 year old Golden Delicious apple trees was performed (Pusey, 1997). Blossoms were treated with either *E. amylovora* CFBP 1430, the mutant 1430^*Y*2*R*^ or PBS buffer (3 mM KCl, 137 mM NaCl, 2 mM KH_2_PO_4_, and 10 mM Na_2_HPO_4_) as mock infection. Racks were cleaned and autoclaved before the experiment. Alternating 24 wells per rack were filled with 2 ml H_2_O. The wells were sealed with scotch tape, which was perforated. Stems were cut freshly to ensure water uptake before transferring the blossoms into the filled wells. Bacteria grown overnight on LB plates were scratched off and resuspended in PBS. OD_600 *nm*_ was adjusted to 1.0 and a 1:50 dilution was performed to generate approx. 10^7^ CFU/ml. A total of 20 μl bacterial suspension or PBS were pipetted directly onto the receptacle. The racks were transferred into storage boxes (5 l) which were laid out with paper towels soaked with 100 ml H_2_O to ensure humidity. The blossoms were incubated at 26°C for 4–5 days before scoring according to an adjusted rating system (Llop et al., 2011). Healthy blossoms without disease symptoms are classified as grade 1. Visible symptoms on the blossom (browning of the calix) were referred to as grade 2. Blossoms with disease symptoms in the calix and the stipe of the blossoms corresponded to grade 3 (Zengerer et al., 2018). The experiment was performed three times.

### Whole Genome Sequencing

Identification of genetic modifications in 1430^*Y*2*R*^ was achieved by whole genome sequencing. DNA was extracted as described previously (Born et al., 2011) and sheared into 550 bp fragments on a Covaris E220 (Covaris, Woburn, MA, United States). Libraries were prepared on an Illumina NeoPrep System (Illumina, San Diego, CA, United States) using a TruSeq Nano DNA kit (Illumina) with six PCR cycles according to manufacturer’s instructions. Paired-end 300 bp sequencing was performed on a MiSeq instrument (Illumina) using a 600-cycle MiSeq Reagent Kit v3 (Illumina) following manufacturer’s instructions. Mapping of the reads was done against the earlier published genome of *E. amylovora* CFBP 1430 [GenBank accession numbers FN434113 (chromosome) and FN434114 (pEa29)] (Smits et al., 2010a) using SeqMan NGen v12 (DNASTAR, Madison, WI, United States) and checked for single nucleotide polymorphisms (SNPs) using the DNASTAR Lasergene package subroutine SeqMan.

### Complementation of 1430^*Y*2*R*^ Mutants

The plasmid pBAD18 (Guzman et al., 1995) was linearized with *Eco*RI and *Hin*dIII and purified. The gene with the locus tag *EAMY_2231* and its ribosomal binding site were amplified from *E. amylovora* CFBP 1430 by PCR with Gibson primers (NEBuilder Assembly Tool v1.12.18) EAMY_2231 fw (5′-TGG GCTAGCGAATTCGAGCTCAGGAGGTCGTAATGCATAAGA TCTGCTATTTC-3′) and EAMY_2231 rev (5′-TGCATGCCTGC AGGTCGACTCTAGACTATATTAATTCGTTATAGGCGG-3′) using the KAPA HIFI™ PCR kit (KAPA Biosystems, Wilmington, DE, United States). The PCR product with the correct length was recovered from a 1% (w/v) agarose gel using the DNA Clean and Concentrator™-5 Kit by Zymo Research. The linearized vector pBAD18 and the insert were joined by Gibson assembly (Gibson et al., 2009). The newly formed plasmid was introduced into electrocompetent *E. coli* XL1-Blue cells for amplification. Cells were recovered in SOC medium (2% (w/v) tryptone, 0.5% (w/v) yeast extract, 10 mM NaCl, 2.5 mM KCl, 10 mM MgCl_2_, 10 mM MgSO_4_, and 20 mM glucose) (Hanahan, 1983) and incubated for 1 h at 37°C under vigorous shaking before plating onto LB plates containing 100 μg/ml ampicillin. Correct plasmid insertion was verified by PCR with the primer pair pBAD fw (5′-CTGTTTCTCCATACCCGTT-3′) and pBAD rev (5′-CTCATCCGCCAAAACAG-3′). Correct complementation plasmid pBAD:*EAMY_2231* was extracted with the NucleoSpin^®^ Plasmid Kit (Macherey-Nagel; Düren, Germany) and introduced into electrocompetent *E. amylovora* CFBP 1430 and 1430^*Y*2*R*^. All generated constructs and strains are listed in Table 1.

### Adsorption Assay

To investigate the adsorption of the phages to phage resistant mutants, pull down assays were carried out (Born et al., 2014). *Erwinia amylovora* CFBP 1430 was used as a positive control, sterile LB medium was used as a negative control. Overnight cultures were prepared as described above with the addition of 100 μg/ml ampicillin to maintain pBAD18 plasmids and 0.2% (w/v) arabinose for pBAD18 promoter induction for transformed bacteria. Cells from overnight cultures were washed twice with fresh LB medium and diluted to an OD_600 *nm*_ of 1.0. Ten microliters of Bue1, L1, S2, S6, M7, and Y2 (10^9^ PFU/ml) were added to 990 μl bacteria. After 10 min of incubation at RT under shaking, samples were centrifuged for 5 min at 10,000 × g at 4°C. Unbound phages in the supernatant were quantified using the soft agar overlay method plating on *E. amylovora* CFBP 1430. Phages S6 and M7 could not be studied in pull down assays, because of inconsistent results and lack of proper adsorption. Each sample was tested at least three times independently.

### Amylovoran Quantification

Impact of *EAMY_2231* modifications on amylovoran production was monitored using the amylovoran-cetylpyridiniumchloride (CPC) precipitation assay (Bellemann et al., 1994). Bacteria were grown in MM2 minimal medium composed at 28°C under shaking for 24 h. OD_600 *nm*_ of all samples were adjusted to 1.0 and 1 ml per sample was centrifuged at 10,000 × g for 5 min. A total of 950 μl supernatant of each sample was mixed with 50 μl CPC (50 mg/ml) and incubated for 10 min at RT before OD_600 *nm*_ was measured. As control, the low-EPS producing *E. amylovora* strain Ea4/82 was used. Each sample was tested at least three times independently.

### LPS Extraction and Analysis

Cells were grown overnight in LB medium and washed twice in PBS buffer before the OD_600 *nm*_ was adjusted to 1.0. A total of 1 ml per sample was centrifuged at 8,000 × g for 5 min. The pellet was resuspended in 100 μl SDS sample buffer [90 mM Tris base, 2% (w/v) SDS, 0.02% (w/v) bromophenol blue, 20% (w/v) sucrose, pH adjusted to 6.8 in H_2_O] and boiled for 10 min at 100°C. Samples were cooled down to room temperature before 2.5 μl proteinase K (20 μg/μl) were added. After incubation at 60°C for 1 h, 10 μl of sample were loaded onto an SDS-PAGE gel (12% resolving/4% stacking gel) and let run with 35 A for 2 h. The gels were quickly washed in ultrapure H_2_O (Sartorius, Germany) before being soaked with fresh fixing solution [40% (v/v) ethanol and 5% (v/v) acetic acid in H_2_O] for 1 h. The fixing solution was subsequently replaced with fresh oxidizing solution (fixing solution supplemented with 30 mM periodic acid) for 5 min. After the incubation, the gels were washed 3–5 times with at least 500 ml of ultrapure H_2_O for 15 min to completely remove the oxidizing solution. The gels were soaked in freshly prepared staining solution [1.5 ml 33% (w/v) ammonium hydroxide solution, 14 mM NaOH, 0.5% (w/v) AgNO_3_ in 200 ml H_2_O] for 15 min. After washing the gels three times in ultrapure H_2_O, gels were developed with freshly prepared developer solution (200 ml ultrapure H_2_O supplemented with 50 mg citric acid and 100 μl formaldehyde solution) until bands appeared. The development was stopped with several charges of ultrapure H_2_O.

## Results

### Spontaneous Y2 Resistance in CFBP 1430 Generates Cross Resistance

Spontaneous resistance development in *E. amylovora* CFBP 1430 was tested for the phages Bue1, L1, M7, S2, S6, and Y2. Resistant bacteria were generated for all six phages. The observed resistances against Bue1, L1, M7, S2, and S6 were all shown to be transient. Colonies were again phage sensitive after the phages were eliminated, e.g., not detectable anymore from the culture. Bacteria exposed to Y2 spontaneously generated permanent phage resistant colonies. Even in the absence of Y2, the resistance was maintained. The strain termed 1430^*Y*2*R*^ could be passaged several times without losing the phage resistance trait. Cross resistance of 1430^*Y*2*R*^ against other phages was tested by spotting phage dilutions on 1430^*Y*2*R*^ lawns. Indeed, the 1430^*Y*2*R*^ strain could no longer be infected by Bue1. In addition, reduced efficiency of plating for the two phages L1 and S2 was observed. M7 and S6 infectivity was not affected by the 1430^*Y*2*R*^ modification (Table 2).

**TABLE 2:** Plaque formation of six phages on the generated mutants – symbolizes no plaque formation.

LB	CFBP 1430 (wt)	CFBP 1430 [pBAD18] (vector control)	CFBP 1430 [pBAD18:*EAMY_2231*]	1430^*Y*2*R*^	1430^*Y*2*R*^ [pBAD18:*EAMY_2231*]
M7	++++	++++	++++	++++	++++
S6	++++	+++	+++	+++	+++
Bue1	++	++	++	–	++
L1	++++	++++	++++	++	++++
S2	++++	++++	++++	++	++++
Y2	++	++	++	–	++
**LB Ara 0.2%**	CFBP 1430 (wt)	CFBP 1430 [pBAD18] (vector control)	CFBP 1430 [pBAD18:*EAMY_2231*]	1430^*Y*2*R*^	1430^*Y*2*R*^ [pBAD18:*EAMY_2231*]
M7	++++	++++	++++	++++	++++
S6	+++	++++	++++	+++	+++
Bue1	+++	+++	++	–	++
L1	++++	++++	++++	+++	++++
S2	++++	++++	++++	+++	++++
Y2	+++	++	++	–	++

*Visible plaques are indicated by +. ++ indicate plaque formation visible on the dilution 10^–2^ and 10^–4^. +++ (up to 10^–6^) and ++++ (up to 10^–8^). Plaque formation was tested on LB medium (top) or LB medium amended with 0.2% (w/v) arabinose (bottom). wt, wild type.*

**FIGURE 1:**
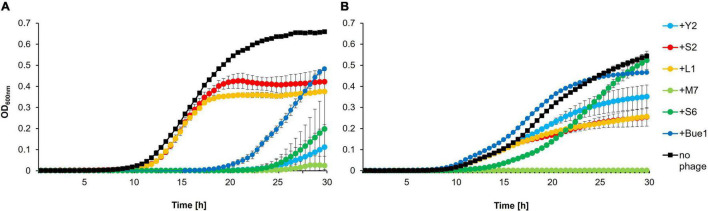
*In vitro* infection assay. Growth of **(A)** *Erwinia amylovora* CFBP 1430 or **(B)** 1430^*Y*2*R*^ infected with phages. Bacterial concentrations of 10^5^ CFU/ml were infected with 10^8^ PFU/ml phage and incubated over 30 h. Optical density (OD_600 *nm*_) was measured regularly at 30-min intervals. Error bars indicate standard deviations. The experiment was performed with three biological and three technical replicates.

Additionally, *in vitro* infection assays were carried out to verify possible cross-resistance (Figure 1). Growth of the wild type strain *E. amylovora* CFBP 1430 incubated with each of the six phages was compared to the growth of 1430^*Y*2*R*^ in the presence of each phage. Y2 had a strong impact on wild type CFBP 1430 cells, preventing regrowth for 17 h. The mutant 1430^*Y*2*R*^, however, was able to induce exponential growth in the presence of Y2. Nevertheless, weaker growth was observed for 1430^*Y*2*R*^ incubated with Y2 (max. OD_600 *nm*_ < 0.4) compared to 1430^*Y*2*R*^ without Y2 (max. OD_600 *nm*_ > 0.5). Incubation with Bue1 generated similar results as the Y2 incubation. Bue1 is able to prevent wild type growth for up to 10 h, whereas no effect was observed, when incubated with 1430^*Y*2*R*^. L1 and S2 infectivity was observed to be weakly in 1430^*Y*2*R*^. In contrast to the spotting assays, the S6 infectivity was strongly impacted in the 1430^*Y*2*R*^ mutant. When S6 was incubated with the wild type, the phage was able to maintain the cell counts below detection level for up to 14 h before regrowth was observed. When mixed with the 1430^*Y*2*R*^ mutant, however, the strain was able to regrow after 5 h. Both strains were successfully infected and controlled by M7. This result suggests that infectivity of Bue1 and Y2 is completely abolished and L1, S2, and S6 infectivity is reduced in the 1430^*Y*2*R*^ strain. Only M7 infectivity was shown to be unaffected by the modification.

### Spontaneous Y2 Resistance Has Little Fitness Impact

**FIGURE 2:**
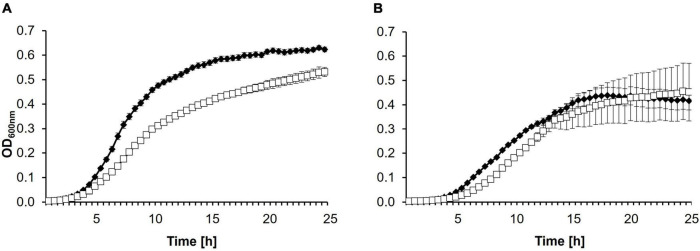
Growth curves of *Erwinia amylovora* CFBP 1430 and 1430^*Y*2*R*^ in **(A)** LB or **(B)** MM2 medium. Cell counts were adjusted to 10^5^ CFU/ml before incubation at 28°C under double orbital shaking for 24 h in a plate reader. Optical density (OD_600 *nm*_) was measured every 30 min. Each strain was measured twice independently with eight replicates per run. Error bars indicate the standard deviation. The experiment was performed with three biological and three technical replicates.

To test whether the mutation in the 1430^*Y*2*R*^ strain has an influence on the fitness of the mutant growth and virulence were monitored. Bacteria were tested for growth defects in LB and MM2 medium (Figure 2). While no difference could be observed in MM2 medium between CFBP 1430 and 1430^*Y*2*R*^, the mutant strain grew weaker in LB medium.

**FIGURE 3:**
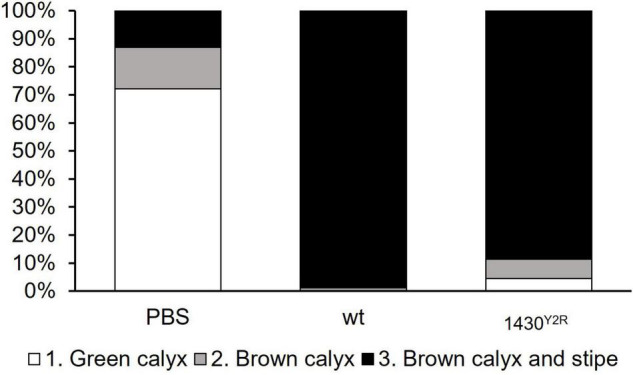
Virulence of 1430^*Y*2*R*^ in detached apple blossoms. Two-year-old Golden Delicious blossoms were infected with 10^7^ CFU/ml bacteria and incubated for 4 days. Read out was performed by evaluating disease symptoms. Grade 1 (green calyx, white boxes) indicates healthy blossoms without disease symptoms. Grade 2 (grey boxes) refers to blossoms with first disease symptoms such as browning of the calix. Blossoms showing advanced disease symptoms on both calyx and stipe correspond to Grade 3 (black boxes). Each treatment was tested at least on 60 individual blossoms. PBS, phosphate buffered saline; wt, wild type. The experiment was performed three times.

Virulence was tested on fresh apple blossoms. As control, the blossoms were mock infected with PBS only. The wild type was able to trigger disease symptoms spreading to the stipe of the blossoms (grade 3) in 98.8% of the infected blossoms. Grade 2 symptoms were observed in 1.2% of infected blossoms. The 1430^*Y*2*R*^ strain generated 88.6% of grade 3 disease symptoms, 6.8% of grade 2, and 4.6% of grade 1 in the infected blossoms (Figure 3).

### *EAMY_2231*, the Source of Phage Resistance

To identify genetic modifications in 1430^*Y*2*R*^ that renders the mutant phage resistant, whole genome sequencing was applied to both the 1430^*Y*2*R*^ mutant and its wild type as control. The whole genome sequencing revealed three single nucleotide polymorphisms (SNP) in 1430^*Y*2*R*^ as compared to the wild type strain. A silent point mutation was found in the gene *rpIV*, encoding the ribosomal protein L22. The gene *ytfB* (cell division protein) contains an adenosine instead of a guanosine at the gene position 472. This mutation results in a glycine to serine transition. The I-TASSER protein structure and function prediction tool (Zhang, 2008) was used to evaluate the impact of the SNP on the predicted structure of YtfB and suggested only minor or no alterations due to the mutation. The third SNP is a nucleotide deletion in the gene *EAMY_2231* (accession no. ON492056). The generated frameshift truncates the protein to 127 amino acids whereby the frameshift affects 42 C-terminal amino acids. The gene *EAMY_2231* is annotated to encode a glycosyltransferase group 1. The encoded protein harbors a GT4_CapM-like glycosyltransferase domain. Proteins encoding such a domain are responsible for transferring UDP-, ADP-, GDP-, or CDP-linked sugars to a variety of substrates and are important in biosynthetic processes. These proteins can be involved in the synthesis of exopolysaccharides or lipopolysaccharide cores (Stingele et al., 1999; Cote and Taylor, 2017). It is hypothesized that the encoded protein *EAMY_2231* could, similarly to *cap1E* in *Streptococcus pneumoniae*, be required for the synthesis of capsular polysaccharides (Muñoz et al., 1997; Marchler-Bauer et al., 2017).

Concluding from these results, the SNP in *EAMY_2231* is the most promising cause for the observed phage resistance. A deletion of the entire gene was attempted. Although different approaches were tested, no mutant could be recovered. A complementation of the complete *EAMY_2231* gene was generated to recover the 1430^*Y*2*R*^ mutation. Complementation of *EAMY_2231* in 1430^*Y*2*R*^ rendered the mutant sensitive again to the phages Y2 and Bue1 (Table 2). The wild type phenotype could therefore be re-established. Furthermore, the complementation of *EAMY_2231* generated comparable infectivity levels to the wild type for L1, S2, and S6 in spotting assays (Table 2).

**FIGURE 4:**
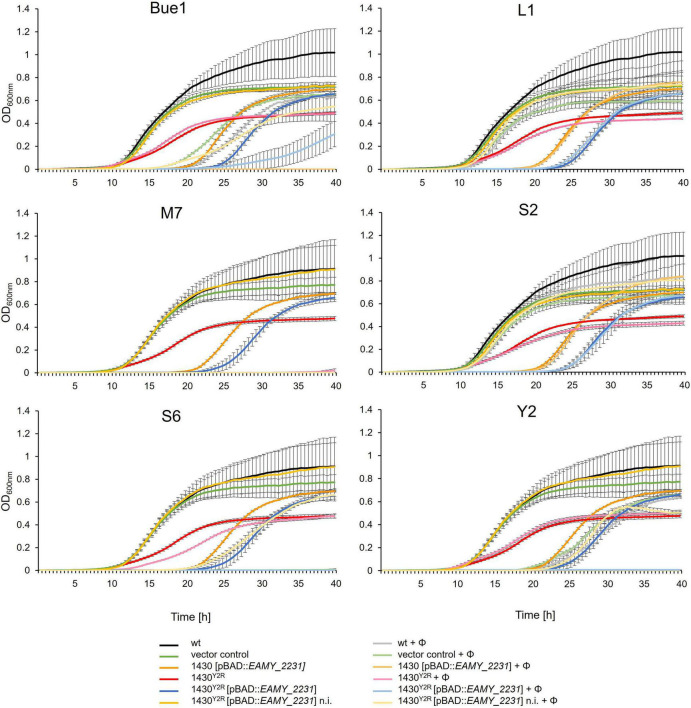
Phage impact on growth of different strains. The wild type (wt), the empty vector control, the overexpression of *EAMY_2231* (1430 [pBAD18:*EAMY_2231*]) the spontaneous Y2 resistant strain 1430^*Y*2*R*^, and 1430^*Y*2*R*^ [pBAD18:*EAMY_2231*] were incubated with the phages (Φ) Bue1, L1, S2, S6, M7, and Y2 or without phages as control. Error bars indicated standard deviation. n.i., not induced. The experiment was performed with three biological and three technical replicates.

The impact of the *EAMY_2231* on the interaction of phages with the host cell were investigated (Figure 4). Generally, the growth of mutants with an induced *EAMY_2231* complementation vector was weaker than the induced vector control and 10 h delayed in the absence of phage. This suggests strong growth deficiencies when *EAMY_2231* is highly expressed. The non-induced complementation in 1430^*Y*2*R*^ [pBAD18:*EAMY_2231*], however, generated comparable results to the empty vector control. This indicates that the used pBAD promotor could be slightly leaky and that *EAMY_2231* is required only in low concentrations to generate the wild type phenotype.

Bue1 was able to successfully infect the wild type strain, the empty vector control, 1430 [pBAD18:*EAMY_2231*], and the non-induced strain 1430^*Y*2*R*^ [pBAD18:*EAMY_2231*]. Weak infection could be observed when Bue1 was incubated with the induced 1430^*Y*2*R*^ [pBAD18:*EAMY_2231*]. Bue1 had no effect on 1430^*Y*2*R*^.

Phages L1 and S2 had no or only a very weak impact on all the tested mutants and complemented strains. Phage M7 was able to completely control all tested mutants for up to 28 h. In the last hour of incubation, a weak regrowth of 1430^*Y*2*R*^ but not of the other strains was observed, suggesting that M7 infectivity might be weakly affected by the mutation found in 1430^*Y*2*R*^. Phage S6 generated similar results as M7. The only exceptions are 1430^*Y*2*R*^ where only weak infectivity was measured and the non-induced 1430^*Y*2*R*^ [pBAD18:*EAMY_2231*] where S6 was unable to control mutant growth after 20 h.

No infectivity was observed when Y2 was incubated with 1430^*Y*2*R*^. In the non-induced 1430^*Y*2*R*^ [pBAD18:*EAMY_2231*] addition of Y2 resulted in a ca. 10 h prolonged lag phase if compared to the same strain without the addition of Y2 indicating that Y2 successfully infected this strain. If gene expression was induced in the presence of Y2 no growth of this strain could be detected. Apparently the combined effect of the inhibitory *EAMY_2231* overexpression and infection by Y2 was lethal.

**FIGURE 5:**
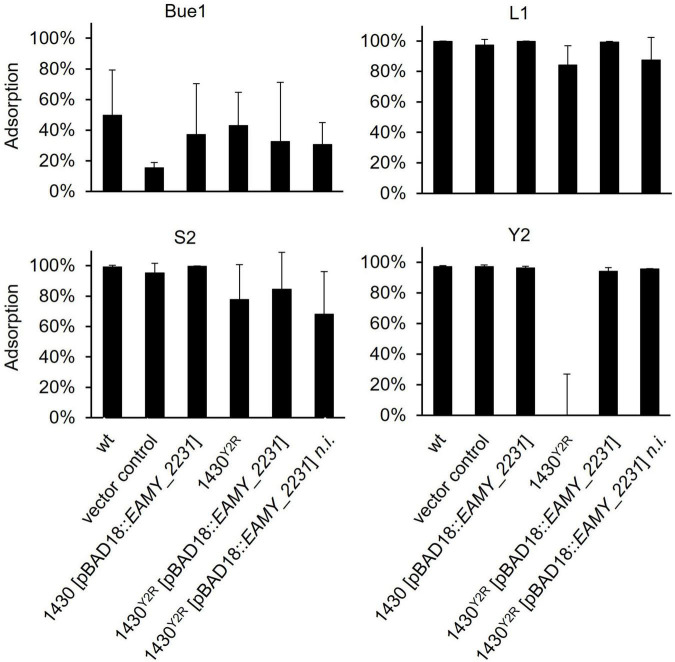
Pull down experiment of all generated mutants applying L1, S2, Bue1, and Y2. The ability of these four phages to bind to the different mutants is illustrated. Error bars indicate standard deviation. Statistical analysis was done by an ordinary one way ANOVA *p* < 0.0001. Each sample was tested at least three times independently. wt, wild type; n.i., not induced.

To test whether the generated modifications have an effect on phage adsorption, pull down assays were carried out (Figure 5). Phage L1 showed stable binding toward the empty vector control, the overexpressed *EAMY_2231* mutant (1430 [pBAD18:*EAMY_2231*]) and the complemented 1430^*Y*2*R*^ (1430^*Y*2*R*^ [pBAD18:*EAMY_2231*]). A reduced adsorption of L1 was observed to the 1430^*Y*2*R*^ strain and the non-induced complemented mutant. Similar results were obtained for phage S2. Phage Y2 was unable to bind to the 1430^*Y*2*R*^ mutant. Complete adsorption was restored in both the non-induced and the induced complemented mutant. No alteration in adsorption was observed in the *EAMY_2231* overexpressed strain 1430 [pBAD18:*EAMY_2231*]. Pull down experiments carried out for Bue1 showed weaker adsorption to the wild type compared to the other phages tested. Although adsorption was observed to be generally weaker for Bue1, the phage was able to bind comparably to 1430^*Y*2*R*^ and the other tested mutants as toward the wild type. Weaker binding affinity was observed for the empty vector control. Pull down experiments carried out with M7 and S6 did not result in reproducible data and were therefore excluded.

### *EAMY_2231* Affects Both Amylovoran and LPS

**FIGURE 6:**
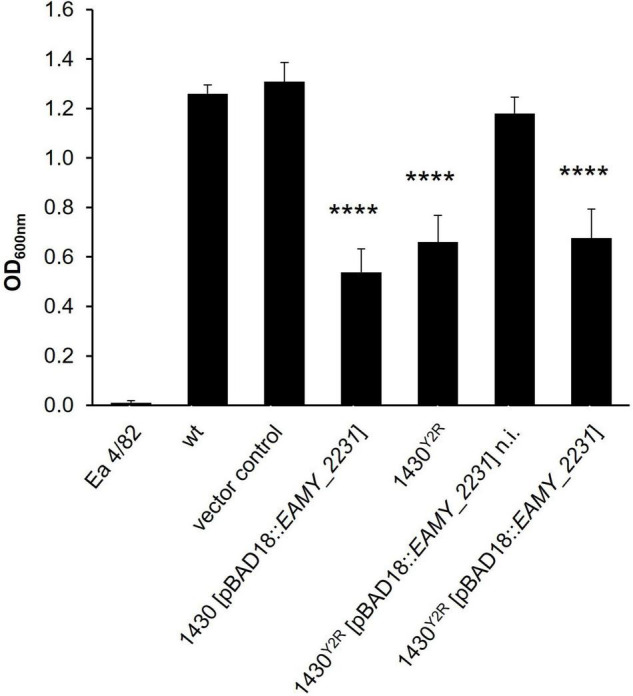
Amylovoran-CPC precipitation. Quantitative amylovoran measurements were carried out for the generated mutants. Produced amylovoran was precipitated with CPC and measured by optical density (OD_600 *nm*_). The EPS lacking strain Ea4/82 was used as negative control alongside wild type CFBP1430 (wt) and CFBP1430 carrying the empty vector (vector control) as positive controls. Error bars indicate standard deviation. Statistical analysis was done by an ordinary one way ANOVA comparing samples to the wild type *p* < 0.0001. Each sample was tested at least three times independently. n.i., not induced.

The amylovoran levels of the generated mutants were tested by amylovoran-CPC precipitation. As control, the low EPS producing strain *E. amylovora* Ea4/82 was used. Compared to the wild type and the empty vector control, a clear reduction in amylovoran production could be observed in the induced strain 1430 [pBAD18:*EAMY_2231*] and the 1430^*Y*2*R*^ strain (Figure 6). The non-induced complementation of *EAMY_2231* in 1430^*Y*2*R*^ restored amylovoran production to wild type level. However, the induced complemented mutant generated similar amylovoran amounts as the 1430^*Y*2*R*^ strain. These results suggest a connection between *EAMY_2231* and amylovoran production.

**FIGURE 7:**
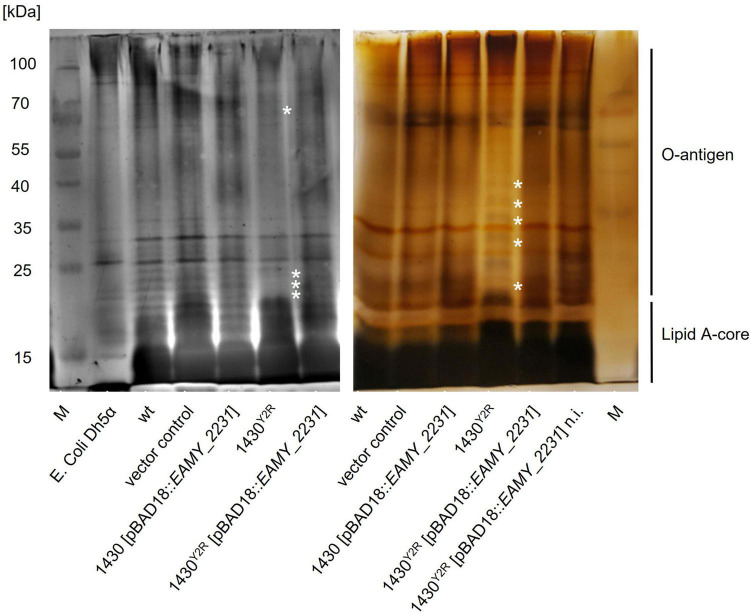
SDS-PAGE analyses of extracted LPS. LPS of the wild type (wt), the empty vector control, 1430 [pBAD18:*EAMY_2231*], the 1430^*Y*2*R*^ strain, and the non-induced (n.i.) and induced complementation in 1430^*Y*2*R*^ [pBAD18:*EAMY_2231*] were silver stained to analyse alterations of the LPS banding pattern. Image of the left gel was taken by an Azure biosystems C300, whereas the image on the right was taken by a standard photo camera. The asterisk indicates changes in the LPS banding pattern of the 1430^*Y*2*R*^ strain.

LPS extraction and analysis was carried out to verify if the *EAMY_2231* mutation results in altered LPS structures. Alterations in the Lipid A and the LPS core of 1430^*Y*2*R*^ could be observed (Figure 7). An altered band pattern is also visible for the intermediate and the long O-antigen section of the 1430^*Y*2*R*^ strain. The complemented 1430^*Y*2*R*^ strain (induced and non-induced) exhibited a comparable band pattern as the wild type, the empty vector control and the *EAMY_2231* overexpressed mutant.

## Discussion

Spontaneous resistance development against phages can occur quickly since bacteria adapt to the selective pressure. Bacteria can apply a collection of strategies to circumvent phage infection. Different stages of the phage infection process can be targeted to generate phage resistance. In this study, *E. amylovora* CFBP 1430 was tested for its potential to induce resistance against six different phages. In the case of Bue1, L1, M7, S2, and S6, the obtained resistance was observed to be transient. Similar findings have been reported for bacteriophages infecting *C. jejuni* (Gencay et al., 2018) and *S. enterica* (Kim and Ryu, 2012). However, after removal of all phages, the resistant bacteria quickly reverted to a phage sensitive state. Likely, this is a form of inherent resistance. The underlying mechanism, however, is unknown so far.

*Erwinia amylovora* 1430^*Y*2*R*^ was observed to be permanent phage Y2 resistant and could be passaged multiple times and stored without losing the phage resistant phenotype. In addition, the 1430^*Y*2*R*^ strain was cross-resistant against other phages. Phage Bue1 was no longer able to infect this strain. L1, S2, and S6, although still able to infect, were shown to have a reduced infectivity.

In order to identify possible permanent alterations in 1430^*Y*2*R*^, whole genome sequencing was carried out and revealed three SNPs in the 1430^*Y*2*R*^ genome compared to the wild type. The most promising mutation was the nucleotide deletion resulting in a frame shift in the gene *EAMY_2231*. Sequence similarities of the gene *EAMY_2231* suggest that the encoded protein can be classified as glycosyltransferase family 1 (Smits et al., 2010b). These proteins can be involved in synthesis of exopolysaccharides or LPS core.

*EAMY_2231* is located in close proximity to the *ams* operon required for amylovoran biosynthesis. Additionally, the string database (string-db.org) indicates interactions between *EAMY_2231* and the proteins encoded by *amsA* (chain length determinant protein) and *amsH* (polysaccharide export protein) both involved in the amylovoran biosynthesis (Bugert and Geider, 1995; Smits et al., 2010a). This suggests an impact on amylovoran production in 1430^*Y*2*R*^. The generated amount of amylovoran in the mutants was measured and a reduction in amylovoran in 1430^*Y*2*R*^ could be observed. This reduction could account for the slightly reduced adsorption of L1 and S2 to the mutant strain and the reduced efficiency of plating, since both phages require amylovoran for host recognition. The complementation of the gene *EAMY_2231*, however, could only establish similar amounts of amylovoran as the wild type when the promoter was not induced. The induction of *EAMY_2231* in the complemented 1430^*Y*2*R*^ and the overexpression of *EAMY_2231* in the wild type generated reduced levels of secreted amylovoran similar to the 1430^*Y*2*R*^ mutant. *EAMY_2231* might be involved in different metabolic processes, which account for the reduction of amylovoran when *EAMY_2231* is overexpressed. The current state of knowledge is that amylovoran is tightly linked to virulence on apple blossoms (Ayers et al., 1979). Although the secreted amylovoran amount in the phage resistant 1430^*Y*2*R*^ is reduced, virulence was shown to be unaffected, indicating that the amount and composition of the produced amylovoran still is sufficient to trigger full disease outbreak in blossoms. Notably, a complete deletion of *EAMY_2231* could not be realized and overexpression of *EAMY_2231* resulted in strong growth deficiencies of *E. amylovora*. In non-induced complemented mutants background expression from the pBAD plasmid was sufficient to complement the phenotype. This indicates that *EAMY_2231* is an essential gene for *E. amylovora* and that is likely only weakly expressed in the wild type.

The phages S6 (Knecht et al., 2022) and M7 (unpublished results) rely on cellulose and the cellulose synthase complex for host cell infection. Cellulose production was monitored and 1430^*Y*2*R*^ produced similar amounts of cellulose as the wild type strain (data not shown). This explains why M7 can still successfully lyse the mutants. S6, on the other hand, was observed to have a reduced infectivity *in vitro*. These findings suggest that aside from cellulose or the cellulose synthase operon, correct *EAMY_2231* function is required for optimal S6 infectivity.

The phages Y2 and Bue1 were unable to infect 1430^*Y*2*R*^. Both Y2 and Bue1 are suggested to recognize their host cells through particular LPS structures. This strongly indicates that *EAMY_2231* is involved in LPS biosynthesis. Three genes in close proximity of *EAMY_2231*, namely *epsF, rfbB1* and *rfbA1*, are annotated to be involved in LPS biosynthesis (Smits et al., 2010b). *rfbB1* and *rfbA1* are supposedly involved in the O-antigen export system as ABC transporter. The gene *epsF* encodes a glycosyltransferase family 8 and is suggested to be required for LPS biosynthesis. The string database^1^ revealed a protein-protein interaction of RfsA and RfaI, both homologous proteins of *epsF* and *EAMY_2231*, respectively, in *E. coli* K12 (Babu et al., 2021). Therefore, the produced LPS was controlled for modifications in the generated bacterial strains. Indeed, strong alterations in the band pattern of 1430^*Y*2*R*^ could be observed. The LPS structure of 1430^*Y*2*R*^ will be further analyzed in detail to identify the exact modification that renders the strain phage resistant. Uncovering the exact LPS structure of 1430^*Y*2*R*^ will reveal the particular receptors recognized by Y2 and Bue1.

On the other hand, resistance of 1430^*Y*2*R*^ against Y2 and Bue1 seems to be mediated by different mechanisms. The resistance against Y2 is clearly achieved by modification of the phage receptor. Pull down experiments revealed that Y2 is unable to adsorb when *EAMY_2231* is truncated. Complementing *EAMY_2231* in 1430^*Y*2*R*^ fully recovered the binding affinity of Y2 to wild type levels. We hypothesize that *EAMY_2231* positively regulates Y2 infection by directly contributing to Y2 receptor synthesis. Bue1 on the other hand could adsorb to both the wild type and 1430^*Y*2*R*^ comparably. The truncation of *EAMY_2231* is therefore not affecting the binding affinity of Bue1. Although Bue1 is able to bind to both the wild type and 1430^*Y*2*R*^, the phage is unable to lyse the latter, indicating that the mutant must use an alternative strategy to resist Bue1 infection. Concluding these observations, we hypothesize that *EAMY_2231* directly affects Y2 binding. Bue1 does not require *EAMY_2231* for adsorption but rather for successful infection in a yet unknown fashion.

Phage resistance can occur at different stages. Since 1430^*Y*2*R*^ affects *EAMY_2231*, it is unlikely that the R-M system or the Abi system are responsible for the resistance development against Bue1 in 1430^*Y*2*R*^. The lack of incorporated Y2 DNA in 1430^*Y*2*R*^ excludes the possibility of sie or CRISPR/*Cas* involvement. As the gene responsible for the resistance has strong similarities to a glycosyltransferase resistance against Bue1 in 1430^*Y*2*R*^ is probably not intracellularly but at the DNA translocation stage. We propose to perform further experiments to verify if Bue1 DNA can be translocated into 1430^*Y*2*R*^.

Understanding interactions between phages and bacteria and investigating the applied resistance mechanisms toward the phages is important to elucidate and anticipate the observed evolutionary arms-race between phages and bacteria. Here, the gene *EAMY_2231* was identified as main source for the spontaneous and permanent Y2 resistance. The identified silent point mutation in *rplV* and the point mutation in *ytfB* (G_472_S transition) are likely ineffective with respect to phage infection. We could further show that the mutation in *EAMY_2331* affects the reaction of the cell against four other phages. In contrast to another study that described attenuated virulence of *Pectobacterium parmentieri* (former pectinolytic *Erwinia* spp.) after phage resistance due to disturbed LPS assembly, sugar metabolism, or EPS production, the observed phage resistance in 1430^*Y*2*R*^ is only minimally linked to fitness loss in the mutant and does not attenuate the strain. LPS silver staining, phage-sensitivity and gene location suggest that *EAMY_2231* is involved in LPS modification. Additionally, we could show that 1430^*Y*2*R*^ produces lower amounts of amylovoran compared to the wild type. All these results suggest that *EAMY_2231* might be involved in several intracellular processes. A possible explanation for these observations could be the fact that *EAMY_2231* harbors a glycosyltransferase domain. A glycosyltransferase is specialized to transfer a particular sugar residue to a specific acceptor substrate (Lairson et al., 2008). The domain incorporated in *EAMY_2231* could be involved in glycosylating peptidoglycan, LPS and capsular polysaccharides. This might explain why the *EAMY_2231* mutation could have such a profound impact on different phages. We therefore propose that *EAMY_2231* plays a crucial role in mediating resistance or reduced infectivity against multiple phages simultaneously.

## Data Availability Statement

The data presented in this study are deposited in the Genbank repository, accession number ON492056.

## Author Contributions

LK and JP performed the experiments. LK, YB, CP, TS, and LF carried out analysis of the data. LK, YB, CP, JP, TS, ML, and LF designed the experiments, wrote the manuscript, and contributed to the article and approved the submitted version.

## Conflict of Interest

The authors declare that the research was conducted in the absence of any commercial or financial relationships that could be construed as a potential conflict of interest.

## Publisher’s Note

All claims expressed in this article are solely those of the authors and do not necessarily represent those of their affiliated organizations, or those of the publisher, the editors and the reviewers. Any product that may be evaluated in this article, or claim that may be made by its manufacturer, is not guaranteed or endorsed by the publisher.

## Footnotes

1
https://string-db.org/
